# Mast Cells and Innate Lymphoid Cells: Underappreciated Players in CNS Autoimmune Demyelinating Disease

**DOI:** 10.3389/fimmu.2018.00514

**Published:** 2018-03-21

**Authors:** Melissa A. Brown, Rebecca B. Weinberg

**Affiliations:** ^1^Department of Microbiology and Immunology, Northwestern University Feinberg School of Medicine, Chicago, IL, United States

**Keywords:** multiple sclerosis, experimental autoimmune encephalomyelitis, mast cells, innate lymphoid cells, sex-dimorphic autoimmunity, testosterone, meninges, innate immunity

## Abstract

Multiple sclerosis (MS) and its mouse model, experimental autoimmune encephalomyelitis, are autoimmune CNS inflammatory diseases. As a result of a breakdown in the relatively impermeable blood–brain barrier (BBB) in affected individuals, myelin-specific CD4^+^ and CD8^+^ T cells gain entry into the immune privileged CNS and initiate myelin, oligodendrocyte, and nerve axon destruction. However, despite the absolute requirement for T cells, there is increasing evidence that innate immune cells also play critical amplifying roles in disease pathogenesis. By modulating the character and magnitude of the myelin-reactive T cell response and regulating BBB integrity, innate cells affect both disease initiation and progression. Two classes of innate cells, mast cells and innate lymphoid cells (ILCs), have been best studied in models of allergic and gastrointestinal inflammatory diseases. Yet, there is emerging evidence that these cell types also exert a profound influence in CNS inflammatory disease. Both cell types are residents within the meninges and can be activated early in disease to express a wide variety of disease-modifying cytokines and chemokines. In this review, we discuss how mast cells and ILCs can have either disease-promoting or -protecting effects on MS and other CNS inflammatory diseases and how sex hormones may influence this outcome. These observations suggest that targeting these cells and their unique mediators can be exploited therapeutically.

## Introduction to Multiple Sclerosis (MS) and Experimental Autoimmune Encephalomyelitis (EAE)

### MS: A Sex-Dimorphic Autoimmune Disease With a Variable Course

Multiple sclerosis is a CNS demyelinating disease of unknown etiology [reviewed in Ref. (1)]. Although past estimates indicate that this disease affects over 2.5 million people worldwide, more recent studies, reported at the 2017 ECTRIMs meeting, suggest that this is a significant underestimation given that 1 million cases were documented in the US alone^1^. Disease susceptibility is influenced by a combination of environmental and genetic factors that trigger a chronic autoimmune disorder in which myelin-specific T cells gain access to the CNS through the normally restrictive vasculature of the blood–brain barrier (BBB). Here these cells orchestrate inflammatory damage to the myelin-producing oligodendrocytes, the nerve-insulating myelin sheath and the nerve axons. The resulting loss of saltatory nerve conduction leads to variable neurological dysfunction such as muscle weakness and spasm, loss of motor function and cognitive deficits. Multiple forms of MS exist that are categorized on the basis of variable disease progression, the most common form being relapsing-remitting (RR) MS, accounting for ~85% of cases. In RR MS, transient episodes of clinical symptoms are interspersed with periods of complete or partial remittance, although in many cases RR MS transitions to secondary progressive MS that continues to worsen or reaches a plateau.

Like many autoimmune diseases, MS exhibits a female bias (2–4). The incidence of MS is 3 to 4 times higher in women than in men and women exhibit clinical symptoms at an earlier age and more often experience a RR course. In contrast, men are more prone to develop primary progressive disease in later life. The molecular mechanisms that dictate sex-dimorphism are still largely undefined, yet it is clear that interactions between X chromosome dosage, microbiota, environment, and sex hormones all contribute (3, 5). Both male- and female-dominant hormones are implicated in protection. MS symptoms often improve during late pregnancy and correspond to the high levels of estriol, a hormone proposed to dampen the immune response to the “allo” fetus by generating tolerogenic dendritic cells (DCs) (6). The delayed onset of MS and a more severe disease course in men correlates with the physiologic age-related decline in testosterone, a steroid hormone primarily secreted by the testes (7, 8). Testosterone treatment of male patients can improve MS (9, 10). For example, daily testosterone therapy for 12 months reversed gray matter atrophy and enhanced cognitive performance in a cohort of 10 men with RR MS (10).

### EAE: A Rodent Model of MS

Much of what we know about the mechanisms that mediate MS pathogenesis was originally discovered in studies of the rodent model of disease, EAE [reviewed in Ref. (11, 12)]. There are only a few spontaneous models of disease, thus EAE is most often induced by active immunization with myelin proteins or peptides derived from the myelin sheath. EAE can also be elicited by the transfer of encephalitogenic myelin-specific T cells to a naive recipient. The most common models of disease utilize MOG_35–55_-immunized C57BL/6 mice or PLP_139–151_-immunized SJL mice. These mice present with ascending paralysis, and spinal cord involvement is thought to predominate. However, there is significant inflammatory infiltration in the brain with time. In fact immune cell infiltration into the hindbrain precedes the appearance of these cells in the spinal cord in SJL mice. Studies in C57BL/6 mice have been particularly informative because of the many gene deletions placed on this background. Of particular value are the 2D2 mice, which contain a transgene encoding a MOG_35–55_-specific TCR (13). Not only do 2D2 mice facilitate the study of MOG_35–55_-specific responses in EAE but they also provide a useful model to study the spontaneous optic neuritis that occurs in human disease.

SJL mice arguably provide a better model of human disease because these mice develop a relapsing–remitting course that recapitulates the most common form of MS. In addition, similar to humans, there is a marked sex-dimorphism in disease (14). Female SJL mice are more susceptible to EAE, while males, particularly young males, are relatively resistant. There have been considerable efforts to identify the variety of factors that contribute to these sex-determined differences in susceptibility. Studies by Voskuhl and colleagues used the XX and XY^−^ four core genotype mice to demonstrate that X chromosome dosage is a critical susceptibility factor (15). XY^−^ mice lack the *Sry* sex-determining region on the Y chromosome and like XX mice are gonadal females, eliminating the potentially confounding influences of hormones. Adoptive transfer of lymph node cells from PLP_131–159_-immunized XX mice to naïve recipients induced significantly more severe disease that XY^−^ cell transfers implying that XX cells have greater encephalitogenic potential. It is evident that protection in males is not associated with a lack of a myelin-specific T cell response, but rather one that is qualitatively different: whereas females generate a robust encephalitogenic Th1/Th17 response, males produce a Th2 response that is non-pathogenic in this setting (16–19). Differences in basal BBB integrity are also implicated in female susceptibility. SJL females exhibit higher cerebellar expression of the sphingosine-1-phosphate receptor 2 (S1PR2) and signaling through this receptor destabilizes vascular adherens junctions resulting in increased BBB permeability (20).

As in humans, hormones influence EAE susceptibility. Pregnancy is associated with reduced disease symptoms and a link between testosterone and protection has been well established (3, 4). Male SJL mice are more susceptible to disease as they age corresponding with decreasing testosterone levels (21). Testosterone treatment of SJL females attenuates EAE by shifting the pathogenic IFNγ-dominated anti-myelin response to one characterized by the production of IL-4 and IL-10. Expression of other pro-inflammatory cytokines including TNF and IL-1β is suppressed as well (16, 18, 22, 23). Conversely, castration or treatment of male mice with flutamide, an androgen receptor (AR) antagonist, results in increased disease severity (16, 24). Male SJL recipients develop EAE after adoptive transfer of primed T cells from female donors, indicating that testosterone exerts a protective influence during T cell priming (16). However, the precise mechanisms that mediate this testosterone effect have not been completely defined.

## The Meninges are Immune Gateways to CNS Inflammation

**Figure 1:**
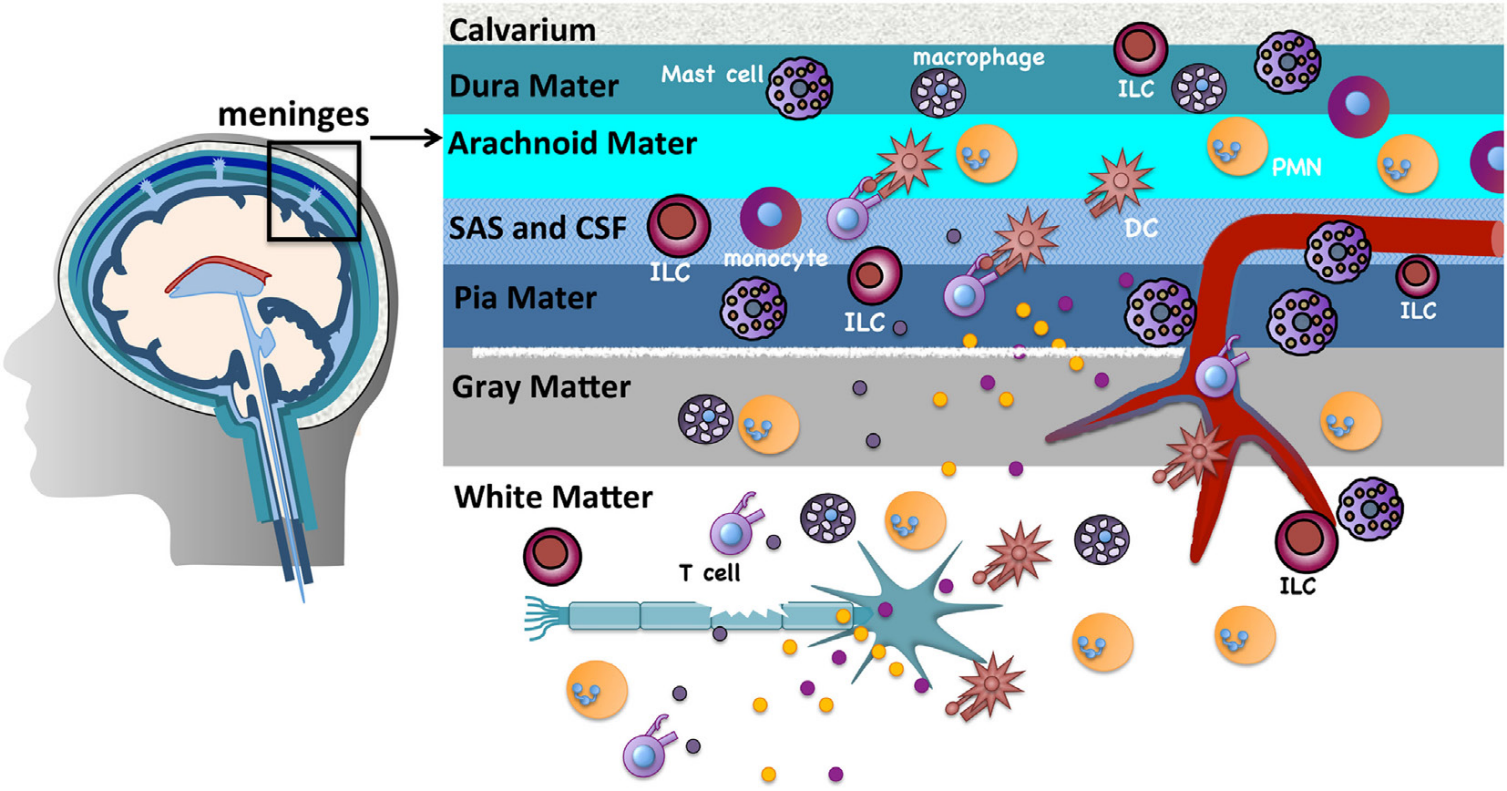
The meninges are sites of active immunity. The meninges are tripartite structures that surround the brain and spinal cord. Multiple innate immune cells reside here. T cells normally transit through the meninges during immunosurveillance and can encounter antigens presented on resident dendritic cells (DCs) or macrophages. Of note, compared to many tissues surveyed, mast cells and innate lymphoid cells are most prevalent in the meninges. These sites serve as first-line protection against infections that threaten the CNS, but are also gateways that can promote chronic CNS inflammation. The inflammatory milieu that is established in the meninges in experimental autoimmune encephalomyelitis/multiple sclerosis is proposed to allow mediator and immune cell infiltration that directly or indirectly damages myelin, olgodendrocytes, and nerves.

The CNS parenchyma is the main target of immune destruction in MS. However, the meninges, highly vascularized tissues that surround the brain and spinal cord and enclose the cerebrospinal fluid (CSF), serve as critical gateways to local inflammation at these sites [reviewed in Ref. (25)]. The meninges are tripartite tissues that are comprised of the outermost dura mater, which lies directly under the skull or vertebral column; the middle arachnoid mater, named for its spider web-like appearance; and the innermost pia mater that often directly adheres to the CNS parenchyma (Figure 1). The arachnoid mater and pia mater are collectively referred to as the leptomeninges. In the human brain, the pia mater follows the extensive involutions of the sulci and gyri thus comprising the largest surface area of the three meningeal layers. CSF drains through the subarachnoid space, an anatomical gap between the leptomeninges. Once thought to serve merely as physical protection for the brain and spinal cord, there is increasing evidence that analogous to other “barrier” sites that interface with the external environment such as the skin, airways, and gastrointestinal tract, the meninges function to provide first-line protection against infections that threaten the CNS. Innate immune cells including DCs, macrophages, mast cells, and innate lymphoid cells (ILCs) are normal residents and circulating cells such as neutrophils and T cells transit through the meninges in the course of normal immunosurveillance (26–28). Importantly, lymphatic vessels were recently discovered within the meninges and likely provide a conduit for CNS fluid, immune cells, and macromolecules to access the meninges and the draining deep cervical lymph nodes (29, 30).

Prior to the onset of clinical symptoms in EAE and several days before inflammatory cells are detected in the CNS, there is an influx of peripherally derived immune cells within the meninges (28). Myelin antigens that drain from the CNS to the meninges *via* the CSF are presented by infiltrating or resident APCs to circulating myelin-specific T cells (27, 31–34). The ensuing T cell reactivation results in a local but sustained meningeal inflammatory response that is characterized by cytokine and chemokine production, the influx of additional T cells, neutrophils, and monocytes, and subsequent loss of BBB integrity. Such meningeal inflammation is also observed in MS patients. In patients with progressive MS, T cell infiltrates are found more densely distributed in the meninges than in the CNS parenchyma (35). Ectopic lymphoid follicles, which sequester antigen and facilitate B and T cell activation, have been observed in the meninges of MS patients proximal to cortical demyelinating lesions and their frequency correlates with disease severity (36, 37).

## Mast Cells and ILCs in the Meninges: Initiators of Chronic CNS Inflammatory Responses in EAE

Immunologists have been aware of the ability of some types of innate immune cells to direct the outcome of B- and T-cell-mediated responses for many years. Cytokines and co-stimulatory molecules expressed by DCs and macrophages, for instance, have established roles in determining T helper cell differentiation fates and B cell isotype switching. Yet, the influence of other classes of innate cells on these processes is not as well defined. Only recently has the potentially potent influence of neutrophils, basophils, and eosinophils been considered in T- and B-cell-mediated diseases, including autoimmune disease. Our laboratory has most recently focused on two additional innate immune cell types that appear to exert profound effects on T cell function in EAE: mast cells and the non-cytotoxic class of ILCs.

Our interest in mast cells in MS/EAE came from several diverse lines of evidence: (1) Mast cells, well known for their ability to regulate vascular permeability, are juxtaposed with BBB vasculature and often are closely associated with nerves (38). They are also found in the meninges and reside within the CNS where they are most abundant in the thalamus and hippocampus (25, 39, 40). (2) These cells express many effector molecules that have been implicated in disease, including molecules that can directly provoke demyelination (41, 42). (3) Mast cells are found in the demyelinating lesions of MS patients as are transcripts that encode mast cell-associated molecules, such as tryptase, histamine, and FcεR1 (43). (4) Tryptase and histamine are elevated in the CSF of some MS patients, suggesting mast cell activation occurs in disease (44, 45). (5) Drugs that block mast cell degranulation or deplete mast cell granules (proxicromil, cyproheptadine, hydroxyzine) reduce EAE severity (41, 46). Over 17 years ago, we reported that mast cells exacerbated MOG_35–55_-induced disease in female (WB X C57BL/6)F1 mice (47). This finding was subsequently verified in female PLP_139–151_-immunized SJL mice (48). Since that time much of our work has focused on understanding how and where mast cells function to amplify disease severity (28, 49–51). Natural killer (NK) cells, a subset of “cytotoxic” ILCs, have been studied for many years in the context of MS and EAE and have been assigned both pathogenic and protective roles [reviewed in Ref. (52, 53)]. Only in the last 3 years have the non-cytotoxic class of ILCs been implicated in EAE (54, 55).

Like NK cells, there is evidence that mast cells and ILCs can exert either pathologic or protective influence on disease and there is still much to be learned about what determines the nature of their actions in particular settings. Below, we provide a brief overview of the multitude of actions of these cells and discuss what is known about their roles in CNS autoimmune demyelinating disease.

## ILC Overview: Potent Modifiers of Immune Responses

Innate lymphoid cells comprise a relatively heterogenous group of innate immune cells that include NK cells, which enter the circulation and migrate through tissues, and the non-cytotoxic ILC subsets, Group 1(ILC1), Group 2, (ILC2), and Group 3 (ILC3), most of which remain in tissues and exert their effects locally [reviewed in Ref. (56, 57)]. All ILCs express CD45 and IL-7Rα and share a common lymphoid cell precursor with T and B cells but are lineage negative (lin^−^), lacking antigen receptors and other cell surface markers that define T cells, B cells, and myeloid cells.

Natural killer cells were first described in 1975 as important players in protection against viruses and tumors, and have been referred to as the innate counterpart to CD8^+^ T cells [reviewed in Ref. (56)]. They are defined in part by the expression of the cell surface marker NK1.1, NKG2D, the transcription factors Eomes and T bet, and IFNγ. A CD56^bright^ population of NK cells has been identified only in humans that appear to limits inflammation (52). Despite sharing a common lymphoid progenitor with non-cytotoxic ILCs, NK cells differentiate through a distinct developmental pathway.

With the exception of lymphoid tissue inducer cells (LTis), members of Group 3 ILCs, the non-cytotoxic subsets were not discovered until the early 2000s (56). Since that time non-cytotoxic ILCs have been implicated in a multitude of protective immune responses, chronic inflammation, fat metabolism, and tissue homeostasis. Although relatively rare in tissues at steady state, ILCs respond quickly and vigorously to a wide range of microbial and environmental activating signals by local proliferation and production of effector cytokines in amounts comparable to Th cells (57). MHC Class II^+^ ILC2s and ILC3s have antigen presentation activity and thus can also directly interact with CD4^+^ T cells (58, 59). ILCs are most abundant in mucosal tissues, common regions of pathogen invasion or colonization, but they are also present in non-mucosal sites such as secondary lymphoid organs, the meninges, and the CNS. Although generally considered tissue resident cells, Huang et al. recently described a unique circulating “inflammatory” ILC2 population induced by IL-25 that shows S1P-dependent trafficking to tissues (60).

CD45^+^ Lin^−^ IL-7Rα^+^ ILC1s, ILC2s, and ILC3s are analogous to Th1, Th2, and Th17 helper cell subsets, respectively, based on striking developmental and functional parallels and similar to T helper cells, ILCs can also exhibit considerable phenotypic plasticity (57). ILC1s are characterized by the expression of the Th1-determining transcription factor T-bet and produce interferon (IFN)-γ a hallmark Th1 cytokine. ILC2s are RORα^+^ and GATA3^high^, both Th2 lineage-determining transcription factors, and express receptors for prostaglandin D2 (CRTh2), and thymic stromal lymphopoietin (TSLPR). They also express ST2, the heterodimeric IL-33 receptor composed of IL1RAcP (IL-1 receptor accessory protein) and ST2 (also known as IL-1 receptor-related protein or IL-1RL1). Upon activation these cells produce the Th2 cytokines IL-4, IL-5, IL-9, and IL-13. The migratory subset of “inflammatory” ILC2s exhibit high expression of KLRG1 and the IL-25 receptor (60). ILC3s are RORγt^+^ and express the hallmark Th17 cytokines IL-17, GM-CSF, and IL-22. Functionally distinct subsets of ILC3s are distinguished by the expression of c-kit and membrane-bound lymphotoxin α1β2 (LTα1β2), which defines LTis, as well as CCR6, CD4, and NKp46. Tbet^+^ ILC3 subpopulations have also been described (55). There are differences between human and mouse ILCs, particularly ILC3s. In mice, two subsets are distinguished by CCR6 expression. CCR6^+^ ILC3s include CD4^+^ and CD4^−^ LTi cells and CCR6^−^ subsets include the NKp46^+^ and NKp46^−^ groups. In humans, all ILC3s appear to be CCR6^+^ and c-kit^+^ but show variable expression of NKp44, another natural cytotoxicity receptor (56).

## Mast Cell Overview: Masters of Immune Regulation

Mast cells are still best known as central players in allergic inflammation, yet these cells are truly multifunctional [reviewed in Ref. (61)]. Mast cells are present at some frequency in most tissues and, depending on their location, demonstrate heterogeneity in the mediators they produce and in cell surface receptors expressed. Thus their response phenotype can vary in a tissue-specific way. Like ILCs, mast cells are most prevalent in tissues that interface with the external environment, the so-called immune border sites, such as the airways, gastrointestinal and genitourinary tract, and skin where they also contribute to first-line protection against pathogens. Perhaps most densely distributed in the meninges, mast cells, together with other resident innate immune cells, are presumed to have a role in limiting infections that threaten the CNS parenchyma [reviewed in Ref. (25)]. However, mast cells can also contribute to the initiation of chronic inflammation that affects the brain and spinal cord.

A hallmark of mast cells is their ability to store pro-inflammatory mediators such as histamine, prostaglandins, leukotrienes, and certain cytokines in cytoplasmic granules. Within minutes of activation through the high affinity IgE-receptor (FcεR1), mast cell degranulation and release of these effector molecules occurs. Newly synthesized mediators that include cytokines and chemokines are also released in a late phase response. The type of response elicited depends on the site of mast cell degranulation and can range from systemic anaphalaxis to local urticaria, angioedema, and allergic rhinitis. However, mast cells can be activated in a variety of other ways and their contributions are not limited to IgE-dependent immediate type hypersensitive responses. Mast cells express many pattern recognition receptors that interact with conserved microbial molecules (pathogen-associated molecular patterns) or danger-signal associated molecules as well as receptors for cytokines, neuropeptides, complement and hormones. The local millieu of cytokines and chemokines within diverse tissue sites can affect many aspects of innate and adaptive immune cell differentiation, effector function, and trafficking. Mast cells also express MHC Class II, Th polarizing cytokines and co-stimulatory molecules providing the ability to directly interact with T and B cells and regulate their activation response (62–65).

## How to Study Mast Cells: Mast Cell Lines and Mast Cell-Deficient Mice

Mature mast cells do not circulate in the blood and are only diffusely distributed in tissues making the study of these cells notoriously difficult. Many experiments have relied on the use of transformed mast cells or mast cell lines derived from bone marrow precursors after culture in IL-3 and SCF, from precursors in the peripheral blood or mature human foreskin-derived mast cells. Mast cells isolated directly from the peritoneal cavity have also been utilized. In 1978 a mast cell-deficient mouse (WB X C57BL/6)F1-*W/Wv* (WBB6 *Kit*^W/Wv^) was first described. This phenotype is the result of two distinct spontaneous mutations located within the “white spotting locus,” termed *W* and *Wv*, in *Kit*, the gene that encodes the stem cell factor receptor c-kit (66). These mutations do not completely ablate c-kit signaling but cause an ~80–90% reduction of activity. Unlike most hematopoietic cells that only require c-kit signals during early development, mast cells have a strict dependence on vigorous and sustained c-kit signaling for their growth and long-term survival. Differences in a phenotype between wild type and *Kit*^W/Wv^ mice indicate a possible role for mast cells and thus this mouse model has provided an invaluable tool for defining mast cell contributions to protective and pathologic immunity [reviewed in Ref. (67)]. However, these mice also present considerable obstacles: *Kit*^W/Wv^ mice are sterile requiring crosses between *W/* + x *W^v^/* + heterozygotes to generate *Kit*^W/Wv^ mast cell-deficient progeny, and such matings result in only 10–20% of mice with the *Kit*^W/Wv^ genotype. Numerous other c-kit-related defects including macrocytic anemia, loss of melanogenesis, neutropenia, and altered gut mobility exist in these mice. Thus to confirm mast cell involvement, *Kit*^W/Wv^ mice must acquire the wild-type phenotype after transfer of bone marrow-derived mast cells (BMMCs), a procedure that selectively reconstitutes mast cell populations but does not correct other c-kit dependent defects.

Other mast cell-deficient mice have since been described that offer some significant advantages over *Kit*^W/Wv^ mice, although the perfect mast cell-deficient mouse model still does not exist. *Kit*^W−sh/W−sh^ mice have a mutation upstream from the *Kit* promoter that interferes with c-kit expression (68, 69). These mice are fertile and on a pure C57BL/6 background, but have increased numbers of splenic mast cell precursors, basophils, and neutrophils, lack melanocytes, and exhibit a time-dependent loss of mast cells (70). *Cpa3*^Cre/+^ mice, described in 2011, contain a transgene encoding Cre-recombinase under the control of the carboxypeptidase 3 (*Cpa3*) promoter (71). High expression of Cre-recombinase in carboxypeptidase 3-expressing mast cell precursors is toxic, causing genomic instability and the ultimate demise of the mast cell lineage at an early stage in development. These so-called “Cre-master” mice also have reduced numbers of basophils, a cell type that shares a common *Cpa3*-expressing precursor with mast cells, but other hematopoietic cells do not appear to be affected. Lilla and colleagues reported that *Cpa-3-Cre;Mcl-1^fl/fl^* mice exhibit a profound reduction in mast cells in most tissues with the exception of the spleen, but are also anemic, have reduced basophils and are neutrophilic (72). Mast cell protease 5 mice (*Mcpt-*5)-*Cre;R-DTA^fl/fl^* mice have a loss of most peritoneal and skin mast cells, but largely retain mucosal mast cell populations (73).

## Mast Cells as Amplifiers of CNS Inflammation

Despite their limitations, *Kit*^W/Wv^ mice on the (WB X C57BL/6)F1 and SJL backgrounds have been extremely informative in our studies of mast cell contributions to EAE. Female *Kit*^W/Wv^ mice of both strains develop significantly less severe EAE than their wild-type counterparts, a phenotype that is reversed by selective mast cell reconstitution (47, 48). Restoration of mast cells to the meninges alone is sufficient for restoring wild-type disease severity, indicating that mast cells residing in these CNS-proximal tissues are a relevant population in regulating disease onset (51). Indeed, meningeal mast cells are activated within a day after active or passive EAE induction and express many pro-inflammatory genes implicated in disease, including *Tnf, Il1b Cxcl1, Cxcl2*, as well as mast cell-specific genes encoding proteases such as *Cpa3, Mcpt2*, and *Tpsab1* (49).

**Figure 2:**
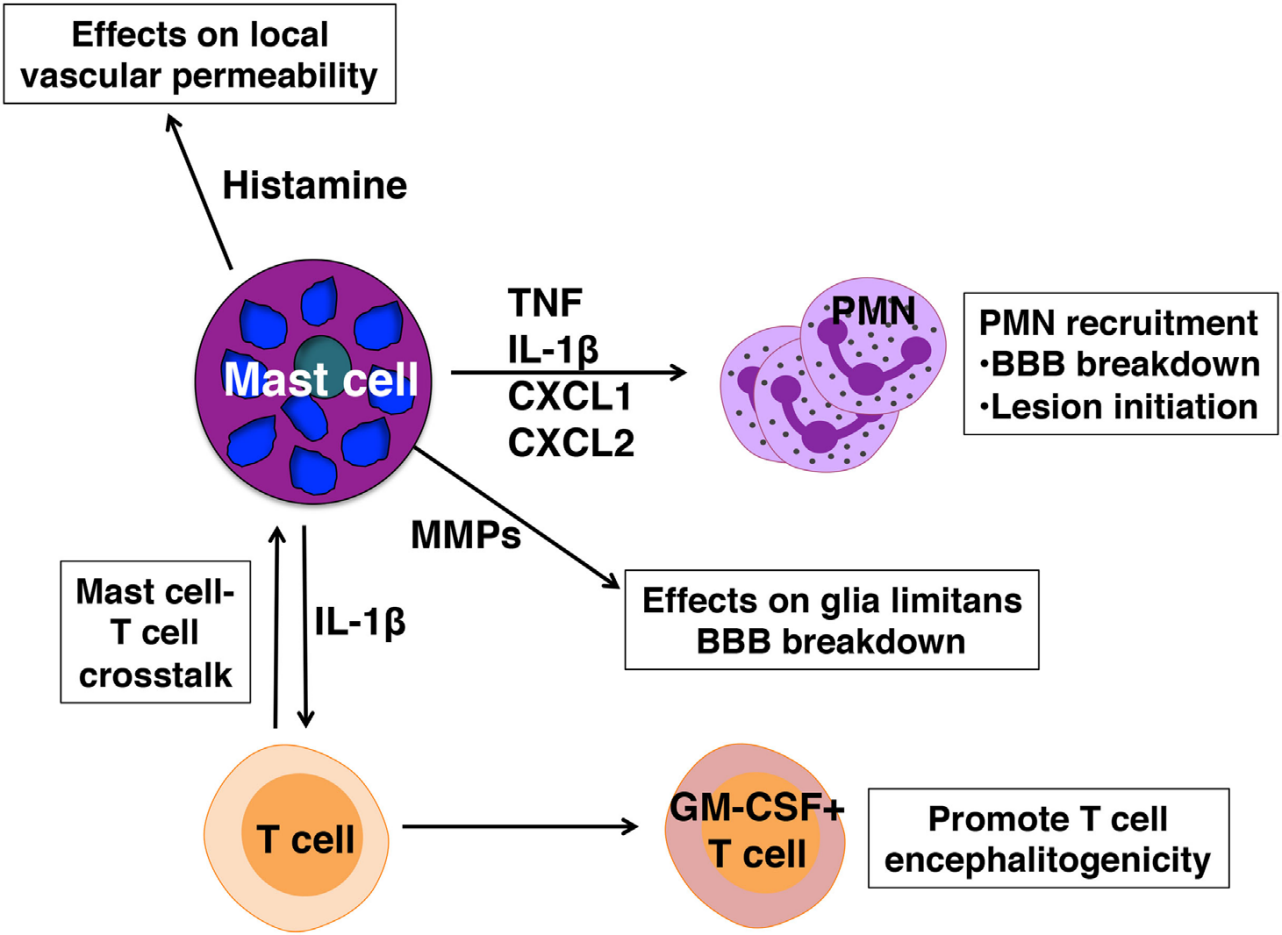
Pathogenic actions of mast cells in experimental autoimmune encephalomyelitis. Mast cells are activated in early disease and express mediators that affect blood–brain barrier (BBB) integrity and license T cells for pathogenicity.

Mast cell-mediated influence on disease severity appears to operate at several levels (Figure 2):

(1) Mast cells located in the meningeal pia mater are situated in close proximity to BBB vasculature and as in peripheral blood vessels, can affect local vascular permeability. In the absence of mast cells frank BBB permeability is not attained (49). As a result, although T cell transit through the meninges is similar in wild-type and *Kit*^W/Wv^ mice, autoreactive Th1 and Th17 cells do not efficiently access the CNS parenchyma in *Kit*^W/Wv^ mice (28, 50).
(2) We have demonstrated that mast cells are essential for the early and relatively robust recruitment of neutrophils to the meninges and CNS, an event dependent on TNF expression by mast cells (28, 49). Neutrophil-related markers, including CXCL1, are increased in the blood at clinical onset of EAE and not only are neutrophils necessary for disruption of BBB integrity (74), they likely have a role in early lesion initiation in the CNS as shown in human studies (75). CXCL5 and neutrophil elastase are elevated in MS patients with active disease and correspond with the presence of acute lesions detected by MRI. Furthermore, plasma levels of CXCL1, neutrophil elastase, CCL2, and CXCL5 in these patients correspond with expanded disability status scale scores, a measure of disease disability.
(3) Mast cell-T cell interactions in the meninges promote T cell encephalitogenicity as well. It has been demonstrated that autoreactive Th17 and Th1 cells primed in peripheral lymphoid organs are not inherently pathogenic, but acquire disease-causing function during their transit to the CNS, a process termed “T cell licensing.” Odoardi et al. showed that myelin-reactive T cells traffic from the lymph nodes through the lungs and acquire new gene expression, including genes encoding molecules that assist in transendothelial migration (76). Our work revealed that interactions between resident mast cells and autoreactive T cells in the meninges induce caspase-1-dependent IL-1β production by mast cells, which in turn elicits T cell production of GM-CSF (50). GM-CSF is a cytokine essential for T cell encephalitogenicity that acts to recruit CCR2^+^ inflammatory monocytes into the CNS (77–80). In the absence of meningeal mast cells or IL-1β production by mast cells, T cell GM-CSF production is reduced, as is EAE severity (50).

Despite evidence in both rodent and human studies that lends credence to the idea that mast cells are critical players in EAE and MS, some experiments with mast cell-deficient mice have not supported such a role [reviewed in Ref. (81)]. There are two striking examples: (1) C57BL/6 *Kit*^W−sh/W−sh^ mice develop more severe disease than their wild-type cohorts (82) and (2) Although *Cpa3*^Cre/+^ mice are refractory to induction of IgE-mediated mast cell disease, no differences in EAE disease severity were observed in side-by-side experiments with wild-type, *CPA3*^Cre/+^, and *Kit*^W/Wv^ mice (71). The reasons for these experimental discrepancies are not clear, but likely reflect the fact that by definition, autoimmune diseases like EAE require T and/or B cells. Thus mast cells can only serve an accessory role. Disease induction conditions that elicit early and strong T cell responses in EAE models, conditions that do not recapitulate the normal evolution of MS in humans, can mask the contributions of mast cells. In support of this idea, Piconese et al. showed that altering disease inducing conditions in *Kit*^W/Wv^ mice changed the apparent mast cell dependence on severe disease development (82). Furthermore, in experiments comparing disease in *Kit*^W/Wv^ and *Cpa3*^Cre/+^ mice early and high morbidity was observed in all groups indicating that a very strong T cell response was elicited during EAE induction (71). The inherent inflammatory state of in *Kit*^W−sh/W−sh^ mice, reflected in increased steady state numbers of neutrophils, may also mitigate the requirement for mast cell-dependent neutrophil recruitment that affects early disease-promoting events.

## *Kit*^w/wv^ Mice Reveal a Mast Cell-ILC2 Connection that Provides Male-Specific Protection in EAE

Studies of EAE in SJL *Kit* mutant mice have also provided insights into the molecular mechanisms that underlie sex-dimorphic EAE in SJL mice. Surprisingly, in contrast to SJL *Kit*^W/Wv^ females, in which the *Kit* mutations protect from severe EAE, male SJL *Kit*^W/Wv^ mice develop severe clinical symptoms (83). They also generate a predominant Th17 anti-myelin response rather than the Th2-dominated response of wild-type males. Importantly, mast cell reconstitution of *Kit*^W/Wv^ males does not restore protection. While this finding cannot eliminate a role for mast cells, it does indicate that another c-kit^+^ cell type is required for protection. Indeed, it was subsequently discovered that *Kit*^W/Wv^ mice have profound defects in ILC2 development and fail to show the increases of ILC2 numbers in the draining lymph nodes, brain, and spinal cord that are characteristic of immunized wild-type males. Because ILC2s are essential for initiating and maintaining Th2 immunity (84, 85), we hypothesized that diminished ILC2s in male *Kit*^W/Wv^ mice are responsible for the lack of a strong Th2 response. These findings raised the possibility that Th17 response-prone females also have defective ILC2 responses. To test this ILC2 numbers in the draining lymph nodes and CNS of PLP_139–151_-immunized males and females were compared and, as predicted, found to be significantly lower in females (19). This muted response is not due to an intrinsic defect in female-derived ILC2s: ILC2 development is comparable in females and males as is the ability of ILC2s to respond to exogenous inflammatory cues delivered both *in vitro* and *in vivo*.

## Decreased IL-33 Expression Limits ILC2 Activation and Promotes Susceptibility in SJL Female Mice

**Figure 3:**
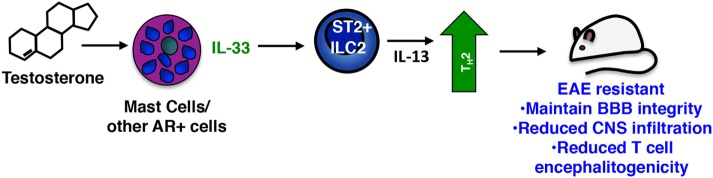
Sex-specific protective actions of mast cells and innate lymphoid cells (ILCs) in male SJL mice. In the sex-dimorphic model of multiple sclerosis in SJL mice, males are protected. Testosterone activates mast cells [and perhaps other androgen receptor (AR)^+^ cells] inducing IL-33, which in turn activates the Th2-promoting action of ST2 + ILC2s. These cells limit the Th17-dominated response characteristic of susceptible females and drive a non-pathogenic Th2 anti-myelin response.

ILC2s can be activated by a number of mediators including TSLP, IL-25, prostagladin D2, IL-7, and IL-33 (86). Among these IL-33, a multifunctional cytokine belonging to the IL-1 superfamily of cytokines, is considered to be the most potent (87). Produced constitutively by epithelial and endothelial cells, DCs, macrophages, and several CNS resident cells, IL-33 is often localized in the nucleus and acts as an alarmin when passively released by damaged or necrotic cells [reviewed in Ref. (88)]. Yet IL-33 is inducibly expressed in certain inflammatory settings through, for example, autocrine activation of P2 purinergic receptors (88–90). We observed that PLP_139–151_-immunized SJL females exhibit significantly reduced induction of *Il33* in multiple tissues when compared to male mice, providing an explanation for their reduced ILC2 activation response. Collectively our data suggest that, similar to its actions in helminth infections and allergic responses (86), IL-33 signals ST2^+^ ILC2s to produce IL-13 and other Th2 polarizing cytokines, which in turn promote a Th2-dominated response in males, one that is non-pathogenic in the context of EAE (Figure 3). It is likely that other ST2^+^ cells are targets of IL-33 and act in concert with ILC2s to confer male-specific protection. Mast cells and basophils also produce Th2 cytokines when stimulated with IL-33 (88) and an ST2^+^ T regulatory cell subset has been defined that demonstrates an IL-33-dependent ability to limit inflammation in a model of inflammatory bowel disease (91). Notably, Matejuk et al. show that decreases in Foxp3^+^ Tregs correspond with declining testosterone levels in aging C57BL/6 mice with severe EAE, although IL-33 was not measured in these studies (24).

The critical role of IL-33 in EAE resistance was confirmed in experiments in which administration of IL-33 to female mice prior to immunization elicits ILC2 activation and prevents disease (19). Strikingly, even when given at the peak of clinical symptoms, IL-33 prevents relapse by inducing ILC2 activation in the meninges and CNS and converting an established Th17-dominated response to one that is Th2. Antibody blockade of IL-33 abolishes EAE protection in males.

## A Context-Dependent *Protective* Function of Mast Cells in EAE

It was reported that the mast cell-specific proteases chymase and tryptase generate a mature form of IL-33 with increased ability to activate ILC2s (92). Thus it is intriguing that mast cells are one important source of IL-33 in male SJL mice. *Il33* induction is significantly diminished in the lymph nodes, meninges and CNS of PLP_131–159_-immunized male SJL *Kit*^W/Wv^ mice, a response largely restored by mast cell reconstitution (19). IL-33 protein is also detected in meningeal mast cells. The mechanism of IL-33 release is still unknown, but similar to cytokines such as TNF, IL-33 may be actively transferred to cytoplasmic granules and released through degranulation (93, 94).

The differential IL-33 response in males and females led to the obvious question: does testosterone influence expression? Serum testosterone levels show an early and transient increase in immunized males and male-derived mast cells express higher levels of the AR *ex vivo*. Direct evidence for testosterone actions on IL-33 induction in mast cells come from studies with male- and female-derived BMMCs (19). Although there is no sex-dependent difference in AR expression in these cultures, testosterone selectively induces *Il33* in male-derived bone marrow mast cells. This male-specific response is also observed in IgE- and Mycobacterium-activated cells, and we speculate that testosterone can affect IL-33 production in at least two ways: (1) Upon immunization, increasing serum levels of testosterone directly drive the IL-33 response by mast cells and other AR^+^ IL-33 expressing cells (Figure 3); and (2) Long-term exposure to this hormone may alter the chromatin landscape during mast cell development, poising this locus for expression in males and repressing it in females. Of note, females also express a unique subset of cytokines that are not induced in males, including TNF and IL-1β, after immunization indicating a striking context-dependent effect on mast cell responses likely regulated by hormones.

Pronounced sex-dimorphic EAE susceptibility is only observed in certain strains of mice (14), suggesting the testosterone-driven IL-33/ILC2 pathway that functions to protect in SJL males does not operate in all strains. This could be due to the documented variations in serum testosterone levels, some of which are potentially too low to trigger this pathway (95). A side-by side analysis of serum testosterone levels in resistant SJL and susceptible C57BL/6 males revealed significantly lower levels in C57BL/6 mice consistent with this idea (19). It will be interesting to determine whether this pathway functions in EAE resistant strains in which both sexes are protected, such as BALB/c and if so, why it is not sex dependent. Obviously, other factors such as strain specific differences in mast cell numbers and cytokine responses also may play a role in differing susceptibilities (96, 97).

## Non-Cytotoxic ILCs in EAE/MS Pathogenesis

**Figure 4:**
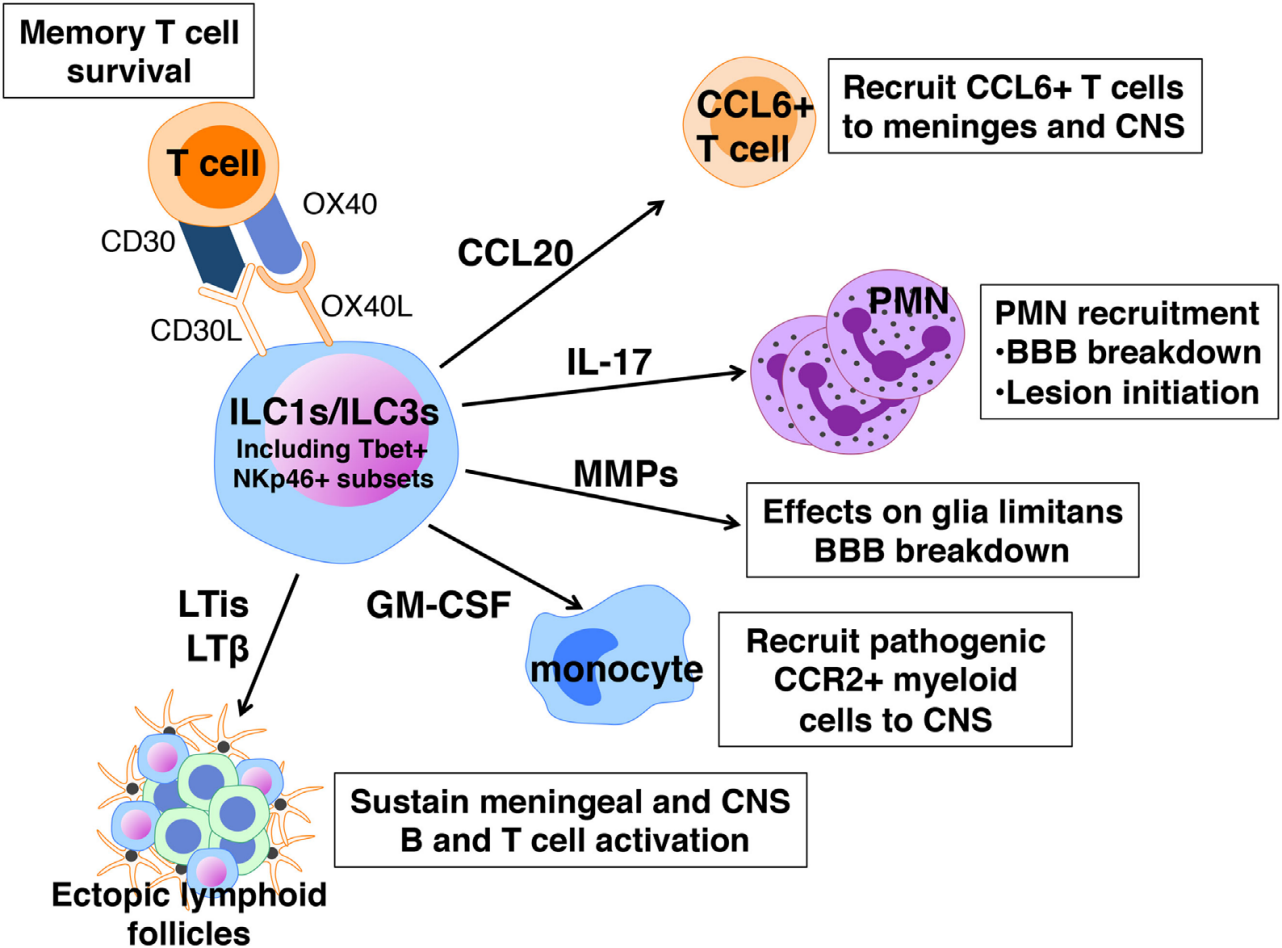
Pathogenic actions of innate lymphoid cells (ILCs) in experimental autoimmune encephalomyelitis. ILC1s and ILC3s express a number of cell surface receptors and mediators that can sustain memory T cells, cause local loss of blood–brain barrier (BBB) integrity, and recruit neutrophils, T cells, and myeloid cells to the meninges and CNS.

In contrast to the apparent protective influence of ILC2s in EAE, ILC1s and ILC3s are implicated in promoting severe EAE. Much of the data to support this claim are correlative and reflect the fact that many features of these cells are consistent with a pathogenic role in disease (Figure 4). Like ILC2s, ILC1s, and ILC3s are normally present in the meninges and CNS and increase in number after disease induction (54, 55). When activated these cells produce IFNγ, IL-17, GM-CSF, and other cytokines that have been linked to EAE pathogenesis. Myelin-specific CD4^+^ T cells with a memory phenotype are the major pathogenic T cell population in EAE (98) and ILC3s express CD30L and OX40L, molecules that promote the maintenance of memory T cell function (99). The LTi subset of ILC3s express relatively high levels of IL-17 and IL-22, as well as membrane-bound LTα1β2 and may also be relevant in disease (100, 101). LTis are critical for the normal development of lymph nodes and Peyer’s patches, functions dependent on LTα1β2, but also drive ectopic lymphoid follicle formation (eLFs) in non-lymphoid tissues. Although eLF formation in response to persistent bacterial and viral infections are protective, these structures, which range from aggregates of diffuse T and B cells to those that resemble conventional lymphoid organs, are hallmarks of the chronic inflammation associated with several autoimmune diseases including MS [reviewed in Ref. (101)]. It has been hypothesized that these follicles form the framework for the sequestration and presentation of autoantigen to T and B cells as well as for intrathecal (subarachnoid) antibody production, another distinguishing feature of MS (102). Notably ectopic lymphoid follicles are found closely associated with inflamed vasculature within the leptomeninges where ILCs reside. Treatment with an antagonist of LTβ blocks the formation of meningeal eLFs and also significantly suppresses EAE onset as well as severity (103).

An incontrovertible role for ILCs in EAE has been difficult to prove because, as for mast cells, there is no selective ILC2-knockout mouse. Mair and Becher were among the first to examine a possible ILC contribution to EAE using a cell depletion approach (104). ILCs, defined as Thy1^+^ Sca1^+^ cells, are increased in the CNS of C57BL/6 mice with EAE. Yet depletion using anti-Thy1 antibodies had no effect on limiting disease severity, suggesting either that ILCs are not important or that Thy1 cell depletion does not target the pathogenic ILC population. Our laboratory observed that adoptive transfer of encephalitogenic Th17 cells to *Rorc^−^*^/^*^−^* mice, lacking both ILC3s and the ability to generate Th17 responses, is not sufficient to induce disease indicating that ILC3s are also required (54). In perhaps the most definitive demonstration of a pathogenic role for ILCs in EAE, Kwong et al. showed that adoptive transfer of MOG_35–55_-specific 2D2 transgenic T cells into mice with a hematopoietic cell-specific deletion in *Tbx21*, are not susceptible to severe disease (55). *Tbx21* encodes T-bet, a transcription factor critical for Th1 differentiation and these findings revealed that in addition to T cells, other Tbet^+^ immune cells are candidates for influencing disease. Using a series of mice with DC- NK- and NKp46-specific deletions in *Tbx21*, the investigators narrowed the critical population to Tbet^+^ NKp46^+^ ILC1s and ILC3s that primarily reside within the meninges. In their absence, T cell influx to the CNS and disease development are significantly reduced. Although the precise mechanism of Tbet^+^ ILCs’ action has not been determined, these cells are purported to function by orchestrating the expression of cytokines, chemokines, and matrix metalloproteinases within the meninges, many of which regulate BBB integrity and cell recruitment to the CNS. For example, the expression of CCL20, which is needed for the entry of CCR6^+^ T cells to the CNS parenchyma and MMPs that break down the glia limitans are much reduced in *Tbx21*^f/f^ NKp46^Cre/+^ mice.

## NK Cells: Another Cell Type with Dual Effects on MS/EAE Outcomes

Natural killer cells have also been implicated in EAE and MS although again, it is unclear whether they exert a beneficial or detrimental role. The answer is probably both give the identification of distinct subpopulations with cytotoxic and “regulatory” functions and cytokine-producing abilities [reviewed in Ref. (52, 53)]. Differences in human and mouse NK cell markers and the lack of selective NK cell-deficient mice limit progress in analyzing these cells in EAE models. CD56^dim^ cells are considered the major cytotoxic NK population and a role in pathogenesis has been proposed due to their ability to activate CNS-infiltrating DCs, assist in Th1 polarization and kill oligodendrocytes, astrocytes and microglia *in vitro*. However, most studies suggest NK cells are beneficial. In mice, cytotoxic NK cells are proposed to “edit” pathogenic lymphocytes and macrophages by direct cytotoxicity or through the production of regulatory cytokines. NK cells produce neurotropic factors such as brain-derived neurotropic factor and neurotropin-3, consistent with a role in neural repair. The most convincing evidence for NK-mediated protection comes from human studies. Immune cell profiling of healthy volunteers and untreated patients with clinically isolated syndrome or RR MS revealed a reduction in the frequency of an NK-like population (CD3^-^ CD56^+^CD8^dim^CD4^−^) in MS patients. Importantly daclizamab therapy (directed to IL-2 receptor α) results in an increase in this population. The increase of a CD56^bright^ subset of NK cells in treated patients, corresponds with the inhibition of contrast-enhancing brain lesions seen in MRI.

## Concluding Remarks

Multiple sclerosis is generally considered to be a T cell-mediated disease although the surprising efficacy of anti-CD20 B cell depleting drugs, such as rituximab, ocrelizumab, and ofatumumab indicate B cells also have an important but as yet unclear role in disease pathogenesis (105). Most targeted therapies have been directed at altering T cell function or migration. The realization that neutrophils, mast cells, and ILCs can exert profound influences on T cell polarization, effector function, and immune cell infiltration to the CNS suggests that new therapeutic strategies should be considered that also target these cells and their mediators. These findings provide strong rationale for studies to continue exploring the T cell modulating roles of mast cells, ILCs and neutrophils in EAE and MS.

Eosinophils and basophils are also worth further examination. Eosinophils are potent cytokine-expressing cells that can serve as APCs and it has been proposed that they may help re-educate pathogenic T cells toward an anergic state (106). In parasitic infections, eosinophils are a major effector cell population and patients with MS who are infected with helminths show less severe disease than uninfected cohorts (107). This observation has been replicated in mice in a study showing that administration of helminth derived products limits EAE severity (108). Protection was dependent on IL-5 and IL-33 and the expansion of eosinophils. Basophils, prolific producers of IL-4 and other Th2 cytokines, are also promising targets. Long considered the “circulating counterpart” of tissue resident mast cells, these (c-kit^−^ FcεR1^+^) cells also have preformed granules that contain a variety of allergic mediators that are released upon IgE cross-linking. Yet the maturation, life span, and unique gene expression profile indicate these cells are distinct (109). Basophils are clearly linked to pathogenesis in a Lyn kinase-deficient mouse model of systemic lupus erythamatosis (110). In this model, development of disease is dependent on the production of IgE autoantibodies. Depletion of basophils limits IL-4 production, reduces IgE, reverses the Th2 bias in these animals and improves the characteristic glomerulonephritis, caused by antibody complex deposition. The strong Th2 cytokine response of basophils would predict that basophils might be protective in MS and EAE and a recent study suggests this may be the case. Anti-FcεR1 treatment depleted basophils and worsened disease in B6 mice and conferred susceptibility to the normally resistant Th2 prone BALB/c mice (111).

There are several lessons to be learned. First, there is now much independent evidence that shifting the pathogenic Th response in MS and EAE to a Th2-dominated one is a viable therapeutic approach and there are a number of drugs currently used in treatment including dimethyl fumerate and glatiramer acetate that have this effect. However, there may be more powerful ways to achieve this outcome by harnessing innate cells; mast cells (in some settings), ILC2s, eosinophils, and basophils, whose physiologic functions in infection are linked to promoting strong Th2 responses. We also need to rethink current therapies that were developed to specifically target T cells but may exert more far-reaching and perhaps unwanted effects. An important example is Fingolimod (FTY720, Gilenya), an S1P receptor antagonist that induces receptor internalization. Because T cells require S1PR signaling to migrate from secondary lymphoid organs to tissues, they are sequestered in the lymph nodes. This drug was also shown to directly affect the S1PR2-expressing BBB endothelium. Blockade of S1P signals increases BBB integrity and limits EAE severity (20). What has not been appreciated is the potential effects of this blockade on other innate immune cells. The circulating KLRG^hi^ “inflammatory” subset of ILC2s are activated by IL-25 and show S1P-dependent migration to tissues (60). Fingolimod treatment abrogated the protective ILC2 mediated responses in a helminth infection model. Thus, although this drug is relatively efficacious in reducing time between MS relapses, inhibition of a Th17-modulating ILC2 response, if shown to be functional in humans, may be undesirable in some patients. Fingolimod also reduces circulating CD56^bright^ NK cells, considered to be protective and such effects may be undesirable (52). In macrophages S1P signaling shifts pro-inflammatory M1s to the anti-inflammatory M2 phenotype, an effect that may be desired in MS (112). Mast cells express S1PRs and are a potent source of S1P and their biology is intimately tied to this signaling pathway [reviewed in Ref. (113, 114)]. It is difficult to predict the effects of Fingolimod treatment on mast cells in the context of MS. Chronic S1P exposure induces the differentiation of a mast cell that is hyperresponsive to IgE-receptor signaling and S1PR2 deficient mast cells show reduced degranulation responses after ATP or phorbol ester/ionomycin activation.

Another class of drugs, receptor tyrosine kinase inhibitors such as imatinib (Gleevac), have been proposed for use in MS treatment (115). Originally used to treat certain cancers, imatinib inhibits the TCR/Abl tyrosine kinase signaling pathway and limits T cell cytokine responses. BCR-Abl-kinase and c-kit signaling are also inhibited by imatinib, suggesting it may be useful to target mast cells in MS. Imatinib blocks mast cell proliferation *in vitro* and attenuates the onset and severity of EAE in both a rat and mouse model of disease (116, 117).

There are several challenges as we go forward. First and perhaps most importantly, we must verify that innate immune cells have similar immunomodulatory effects in human disease. Although the data implicating mast cells and ILCs in EAE is intriguing, there is still is a paucity of information regarding a role for these cells in MS. As discussed earlier mast cells are normally present in the human meninges and brain and are associated with demyelinating lesions. Meningeal tissues from a small cohort of acute MS patients provide evidence of meningeal mast cell-T cell co-localization associated with areas of subpial cortical demyelination (50). Mast cells were also observed in white matter parenchymal lesions of differing demyelinating stages (early active, inactive, and remyelinated), often in close proximity to infiltrating T cells. Even less is known about non-cytotoxic ILCs in MS and this information comes from drug studies in which CD25 blockade reduced numbers of the circulating LTi subset of ILC3s and inhibited meningeal inflammation (118).

In view of the dramatic sex and strain differences in murine responses, we also must consider the possibility that the diverse genetic and environmental backgrounds of humans will lead to variable innate immune cell responses. Thus in some cases we may want to inhibit innate immune cell activation (e.g., in settings where mast cells are pathogenic), while in others, we may want to enhance their activation (e.g., in cases where mast cell production of anti-inflammatory mediators like IL-33 occurs).

Finally, the ultimate goal of all research in autoimmune disease including MS is to develop therapeutic strategies that stop disease progression and confer a lasting cure as well as eliminate the common generalized immunosuppression often associated with current treatments. Our data demonstrating IL-33 can prevent relapses even when administered to mice at the peak of established disease are promising, particularly in view of its actions on multiple cell types. Future studies need to determine whether direct administration of IL-33 to MS patients or use of strategies to enhance endogenous production may be effective in reversing MS symptoms, without dangerous side effects. IL-33 was shown to have direct effects on oligodendrocyte gene expression and induce p38 MAPK phosphorylation these cells, an event linked to myelination (119) indicating that treatment with this cytokine or therapies directed downstream of IL-33 may fulfill a dual role of blocking damaging inflammation as well as promoting myelin repair.

## Author Contributions

MB and RW wrote and edited the manuscript.

## Conflict of Interest Statement

The authors declare that the research was conducted in the absence of any commercial or financial relationships that could be construed as a potential conflict of interest.

## Footnotes

^1^https://urldefense.proofpoint.com/v2/url?u=https-3A__onlinelibrary.ectrims-2Dcongress.eu_ectrims_2017_ACTRIMS-2DECTRIMS2017_199999_mitchell.t.wallin.the.prevalence.of.multiple.sclerosis.in.the.united.states.a.html-3Ff-3Dmedia-3D3&d=DwIGaQ&c=yHlS04HhBraes5BQ9ueu5zKhE7rtNXt_d012z2PA6ws&r=0eACTVfwt5BhCuBiOTML-EGdMefbkdO179LO68_DpLc&m=_ZQOmVYBDgN9vUsahKvcL5WmWp_exI0RGR0YQAI9oMk&s=wAJJCkTxOI3d0lmbRnsSeIWtATJfdHDBauU35HB2-Mg&e=
